# A conserved region within interferon regulatory factor 5 controls breast cancer cell migration through a cytoplasmic and transcription-independent mechanism

**DOI:** 10.1186/s12943-015-0305-5

**Published:** 2015-02-04

**Authors:** Erica Maria Pimenta, Betsy J Barnes

**Affiliations:** Department of Microbiology, Biochemistry & Molecular Genetics, Rutgers Biomedical and Health Sciences, Newark, NJ 07103 USA; Rutgers Biomedical and Health Sciences, New Jersey Medical School-Cancer Center Rm. G1224, 205 South Orange Ave, Newark, NJ 07103 USA

**Keywords:** Interferon regulatory factor 5, IRF5, Epithelial cell migration, Motility, Breast cancer metastasis

## Abstract

**Background:**

Migration of breast cancer cells out of a duct or lobule is a prerequisite for invasion and metastasis. However, the factors controlling breast cancer cell migration are not fully elucidated. We previously found that expression of the transcription factor interferon regulatory factor 5 (IRF5) is significantly decreased as a breast lesion progresses from a non-malignant stage to ductal carcinoma *in situ* and is eventually lost in ~80% of invasive ductal carcinomas examined. Human *in vitro* and murine *in vivo* models of invasive breast cancer confirmed an important role for IRF5 in regulating cell motility, invasion and/or metastasis; yet, the mechanism(s) by which this occurs is not known. Since IRF5 is primarily expressed in the cytoplasm of human mammary epithelial cells, we hypothesized that IRF5 may function in a transcription-independent manner to control intrinsic cell migration.

**Results:**

A series of IRF5 deletion mutants were tested in cell motility, invasion and migration assays. A novel, conserved 10 amino acid domain was identified that regulates mammary epithelial cell migration. This region (∆115-125) is downstream of IRF5′s DNA binding domain and therefore when absent, retains IRF5 transcription activity but loses cell migration control. An IRF5 construct with a mutated nuclear localization signal further confirmed that IRF5 controls migration in a cytoplasmic and transcription-independent manner. Candidate cytoskeletal molecules were identified in MDA-MB-231 cells to interact with IRF5 by immunoprecipitation and mass spectrometry analysis. α6-tubulin was independently confirmed to interact with endogenous IRF5 in MCF-10A cells. Alterations in F-actin bundling after staining EV- and IRF5-231 cells with phalloidin suggests that IRF5 may control cell migration/motility through its interaction with cytoskeletal molecules that contribute to the formation of F-actin networks. Last and most notably, we found that IRF5′s control of cell migration is not restricted to mammary epithelial cells but functions in other epithelial cell types suggesting a more global role for this newly identified cell migratory function of IRF5.

**Conclusions:**

These findings are significant as they identify a new regulator of epithelial cell migration and provide specific insight into the mechanism(s) by which loss of IRF5 expression in mammary epithelial cells contributes to breast cancer metastasis.

## Introduction

Interferon regulatory factor 5 (IRF5) is a member of the IRF family of transcription factors that are expressed in vertebrates. Eleven IRFs have been identified to date (IRF1-11) but only IRF1-9 are found in humans and mice [1-5]. Members of the IRF family share a well-conserved N-terminal DNA binding domain (DBD) comprised of 5 tryptophan repeats spanning the first 115 amino acids [1-5]. In the carboxyl terminus, an IRF association domain (IAD) exists which allows for homo- or heterodimerization with IRFs, STATs and other functional interacting partners, which mediate functional specificity to each IRF [1,3,4,6]. In addition to these conserved domains, IRF5 has two functional nuclear localization signals (NLS), one nuclear export signal (NES) and an autoinhibitory domain (AID) [3,6].

Classically, the transcriptional activity of IRF5 is dependent upon its activation. IRF5 generally resides in the cytoplasm of an unstimulated cell and must translocate to the nucleus for its transcriptional function. Others and we have shown numerous signaling pathways that lead to IRF5 activation and nuclear translocation [5,7-11]. Probably most well known is the induction of IRF5 activation by MyD88-dependent Toll like receptor (TLR) signaling [7,12-14]. In this pathway, ligand bound to receptor induces a cascade of events whereby IRF5 binds to MyD88 and TRAF6 [3,5,13,14], undergoes several post-translational modifications including phosphorylation and possibly ubiquitination [10], and then forms hetero- or homodimers that translocate to the nucleus [4,6,12,13,15,16]. Once in the nucleus, the IRF5 DBD recognizes interferon-sensitive response elements (ISREs) within the promoters of downstream target genes [2,3,17]. Targets of IRF5 are generally pro-inflammatory. IRF5 has been shown to positively regulate cytokines such as Type I interferons (IFNs), interleukin (IL)-6, IL-12, IL-1β, and TNF-α while suppressing IL-10 expression [11,14]. Most studies of this protein to date have been performed in lymphocytes, therefore IRF5 is classically thought of as a transcriptional immune regulator [4,7,11,15].

Although most studies of IRF5 generally assume that activation and nuclear translocation are required for function, there is accumulating evidence that IRF5 does not need to be activated to influence some cellular processes. In many cases, simple overexpression of IRF5 in various cell types can cause a decrease in growth rate, induction of G2-M cell cycle arrest, reduction in colony formation, and an increase in baseline apoptosis [9,16,18-21]. Upon activation of IRF5 in response to DNA damage, death receptor signaling or TLR-induced signaling, only a small portion of IRF5 molecules (10-40%) become activated and translocate to the nucleus, while the rest remain in the cytoplasm [22]. Given previous findings from overexpression assays and the fact that the majority of IRF5 molecules reside in the cytoplasm constitutively [19,23], we hypothesized that IRF5 has some housekeeping or resident cytoplasmic function(s) that is independent of its transcriptional activity.

In support of this hypothesis, loss of IRF5 expression has been observed in many cancers including breast [23,24], lung [25], hepatocellular [26], gastric [27], colon [19] and hematological malignancies [16]. Our lab previously showed that normal breast epithelium expresses high levels of IRF5 and that this expression decreases as malignancy progresses from ductal carcinoma in situ (DCIS) to invasive ductal carcinoma (IDC) [23]. Approximately 80% of IDC patient samples examined to date are IRF5-negative, as well as their corresponding lymph node metastases [23]. In a 3-dimensional (3D) *in vitro* model of invasive breast cancer cell growth, overexpression of IRF5 in MDA-MB-231 cells resulted in a complete reversal of invasive acini outgrowth to normal ductal structure [23]. Additionally, in a xenograft *nu/nu* mouse model using two different breast cancer cell lines made to stably express IRF5, no metastasis was found in mice injected with IRF5-positive tumors compared to metastasis in control cohorts that lacked intratumoral IRF5 expression. IRF5-positive primary tumors were also smaller in number and mass [23]. While IRF5 is known to be immunomodulatory in most cell types, the xenograft studied was done in immunocompromised mice indicating that IRF5 expression in breast cancer cells intrinsically changes their cellular function conferring a less invasive and metastatic phenotype.

In this study, we significantly extend our original findings to further delineate the mechanism(s) by which IRF5 controls breast cancer cell growth and metastasis and ultimately find that IRF5 may be a global regulator of epithelial cell migration.

## Results

### IRF5 expression is a marker of recurrence-free survival in breast cancer

**Figure 1:**
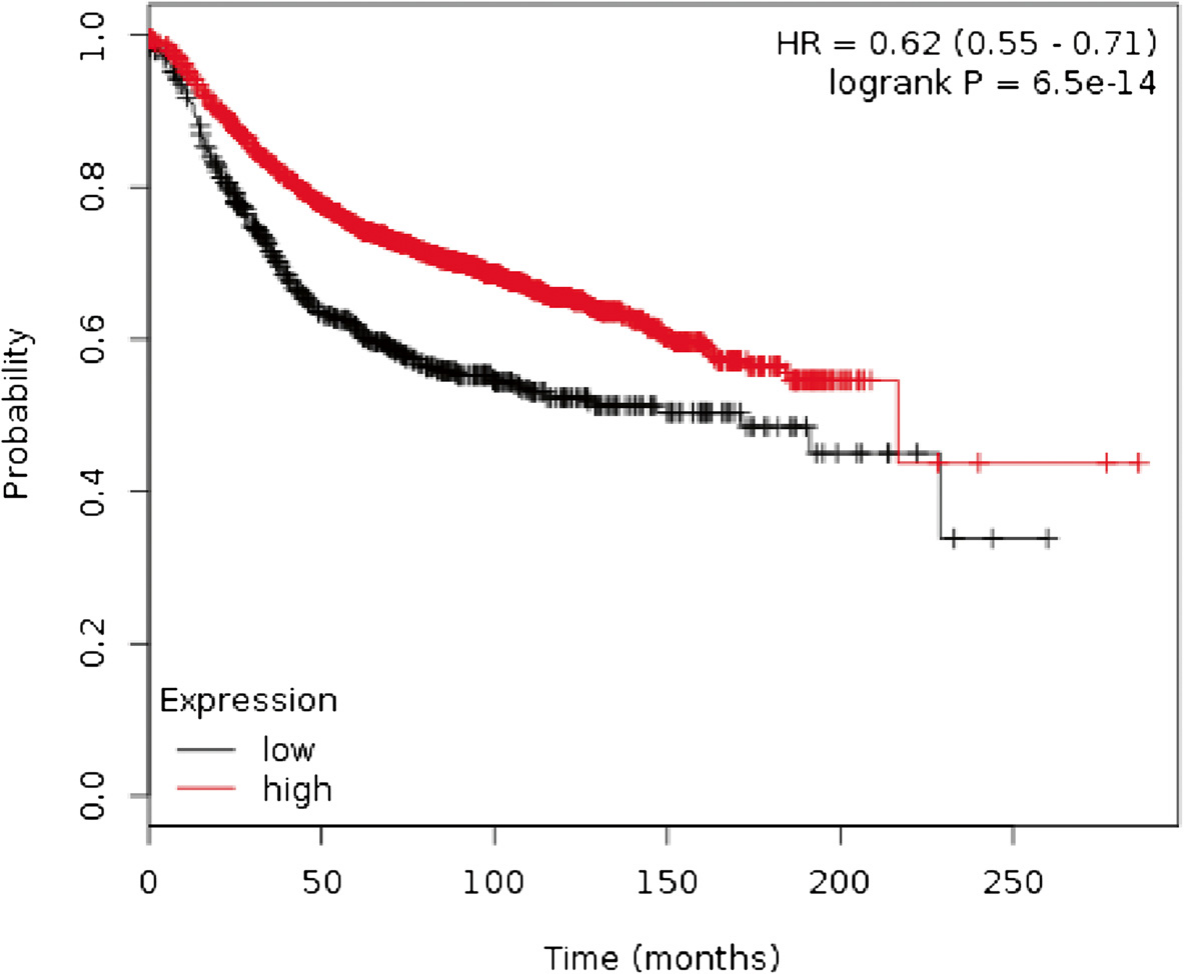
**The lower quartile of** ***IRF5*** **expression is a marker of poor prognosis for recurrence-free survival.** Data are from all primary specimens of breast cancer (n = 3,455) from The Cancer Genome Atlas. Black line indicates low *IRF5* expression, red line indicates high *IRF5* expression. *p* = 6.5 × 10^−14^.

Using data from The Cancer Genome Atlas (TCGA) of all human primary breast cancers (n = 3,455) [28], we performed a correlation analysis with *IRF5* transcript expression and recurrence-free survival (RFS). Data in Figure 1 reveal that the lower quartile of *IRF5* expression is a marker of poor prognosis for RFS (*p* = 6.5×10^−14^). Combined with recent data from screening human breast tumors for molecular signatures that revealed when IRF5 expression is retained, it encompasses a breast cancer signature that predicts increased survival and lower incidence of metastasis [29,30], these data suggest a functional and pathogenic consequence due to loss of *IRF5* expression that relates to human mammary epithelial growth and metastasis.

### IRF5 expression inhibits the migration of breast cancer cells

**Figure 2:**
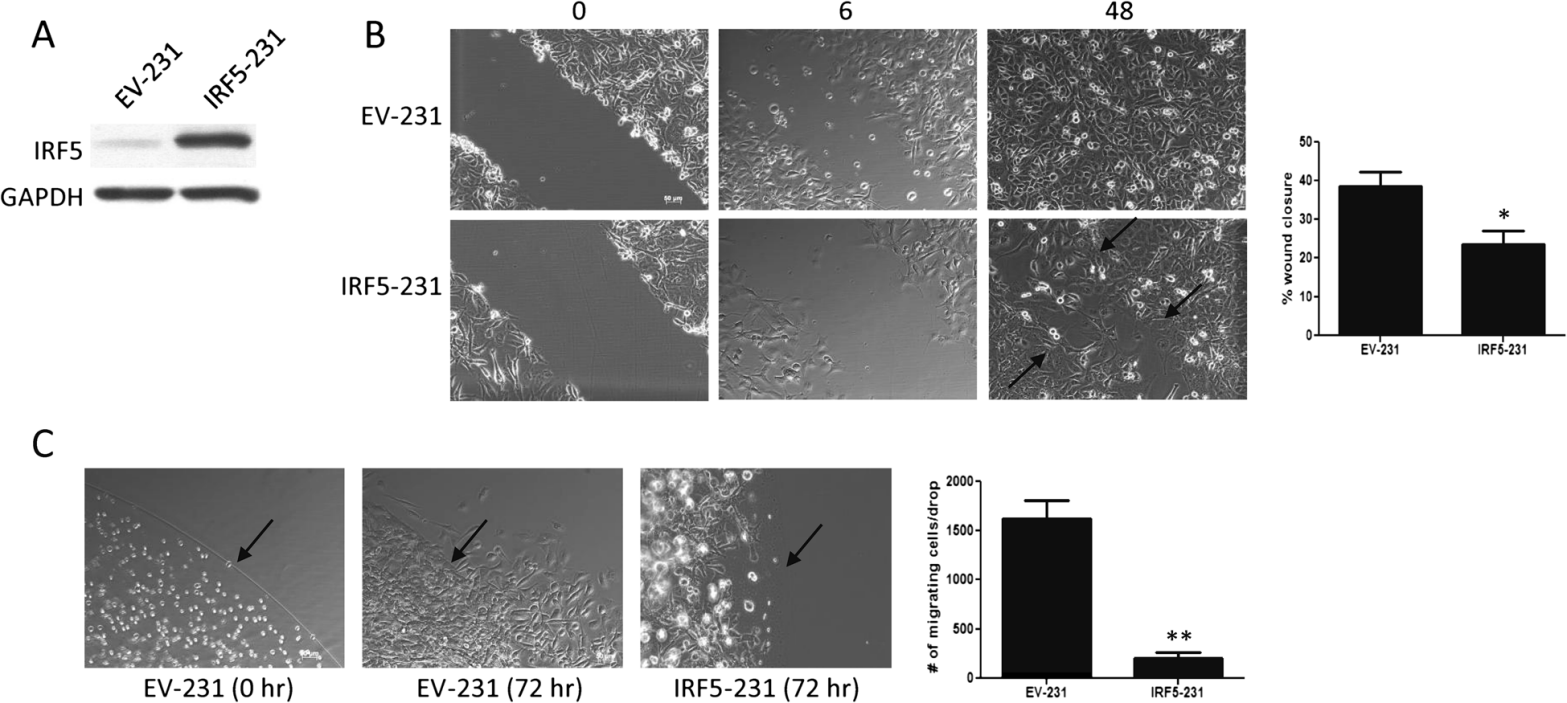
**IRF5 inhibits wound healing and matrigel evasion in MDA-MB-231 cells. A)** MDA-MB-231 cells were retrovirally infected with either empty vector (EV-231) or IRF5 (IRF5-231) expressing pBabe plasmid. Levels of IRF5 and GAPDH protein expression are shown. **B)** Wound healing assays were performed on EV-231 and IRF5-231 cells. Representative pictures are shown from 0, 6 and 48 hours after scratch. Arrows point to a visible wound still present in the IRF5-231 plate at 48 hours post-scratch. Graphical representation of data from the 6 hour time point is shown on the right from at least 3 independent experiments performed in duplicate. **C)** Representative pictures from the matrigel evasion assay are shown. The left-most panel shows EV-231 cells suspended in the matrigel drop at time 0 hours (hr); middle panel shows EV-231 cells escaping the matrigel drop at 72 hrs; right-most panel shows IRF5-231 cells unable to escape the matrigel drop at 72 hrs. Arrows indicate the matrigel drop border. Graphical representation of data from the 72 hr time point is shown on the right. Data are from at least 3 independent experiments performed in duplicate. *p ≤ 0.05, **p ≤ 0.01.

Experimental *in vitro* and *in vivo* data also support a role for IRF5 in mammary epithelial cell migration and metastasis. IRF5 overexpression was shown to revert the highly invasive nature of MDA-MB-231 acini in 3D culture and no metastasis was observed in xenograft mouse models with IRF5-positive tumors [23]. Based on these data, we sought to elucidate the molecular and cellular mechanisms by which IRF5 inhibits cell migration, invasion and/or metastasis. MDA-MB-231 cells were used as the primary cell model as they are highly invasive and express very low levels of endogenous IRF5 [23]. A wound healing assay was performed on MDA-MB-231 cells generated to stably express full-length IRF5 (IRF5-231) versus empty vector control (EV-231) cells (Figure 2A). Data in Figure 2B shows that 6 hours after the wound was created, IRF5-231 cells lagged in wound closure by approximately 20%. At 48 hours, IRF5-231 cells were still unable to completely close the wound as highlighted by the arrows in Figure 2B.

To further characterize the metastatic potential of IRF5-231 cells, we modified an assay previously described by Szymczak et al. that measures the ability of a cell to escape a matrigel drop [31]. Matrigel is a protein matrix composed of collagen and laminin that acts as a model basement membrane. By suspending either EV- or IRF5-231 cells in a matrigel drop and allowing the matrix to solidify, we measured the ability of these cells to degrade a basement membrane as a measure of the invasive potential of the cells. While the EV-231 cells were able to escape the matrigel and grow freely on the tissue culture dish, IRF5-231 cells were never able to escape the protein matrix (Figure 2C). Even up to 5 days, IRF5-231 cells remained within the matrigel drop. These two experiments clearly show that increased IRF5 expression on its own intrinsically reduces MDA-MB-231 metastatic potential.

**Figure 3:**
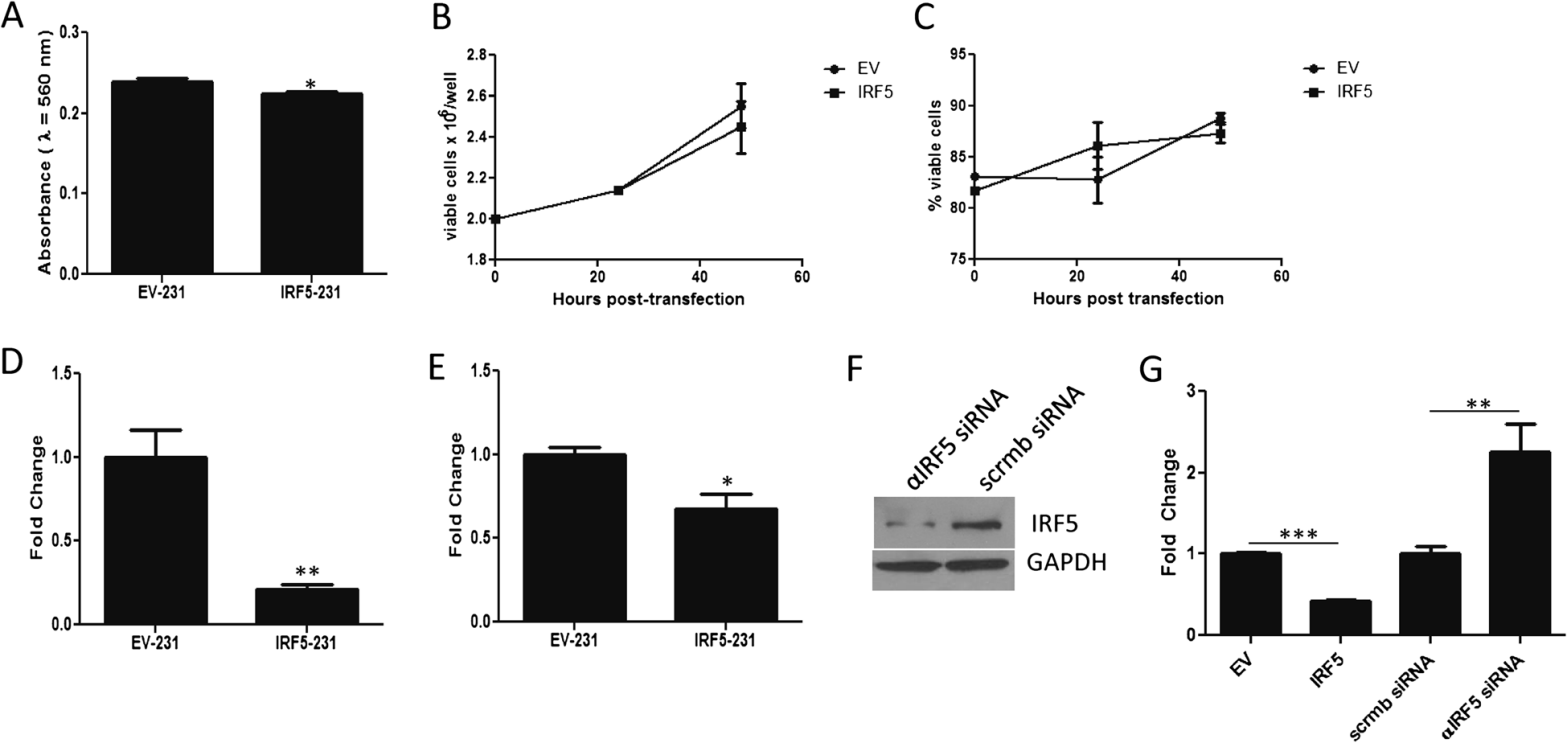
**IRF5 is a major regulator of mammary epithelial cell migration. A)** Graphical representation of data from EV-231 and IRF5-231 MTT assays. **B)** Graphical representation of 231 cell viability at different time points post-transfection. **C)** Similar to **B)** except data shown are percentage of 231 viable cells post-transfection. No statistical difference was found in the number **(A)** or percentage **(B)** of viable cells between EV-231 and IRF5-231. **D)** Graphical summary of data from Boyden chamber assays measuring cell migration to serum-containing media. **E)** Similar to **D)** except data are from Boyden chamber assays measuring cell invasion through matrigel. **F)** Western blot analysis of endogenous IRF5 protein levels in MCF-7 cells transfected with scrambled siRNA (scrmb siRNA) or siRNA targeting IRF5 (αIRF5 siRNA). **G)** Similar to **D)** except the migration assay was performed in MCF-7 breast cancer cells transfected with either empty vector (EV), full-length IRF5 (IRF5), scrmb siRNA or αIRF5 siRNA. All data shown are from at least 3 independent experiments performed in duplicate; *p ≤ 0.05, **p ≤ 0.01, and ***p ≤ 0.0001.

Importantly, in both the wound healing and matrigel evasion assays, cell proliferation, migration and invasion may all play a role in the overall mechanism of IRF5 metastatic inhibition. In order to delineate which function(s) to ascribe to IRF5, a series of assays were performed to individually evaluate each effect. Using the MTT assay to assess proliferation, IRF5-231 cells showed a slight but significant (~6%) decrease as compared with EV-231 cells (Figure 3A). While others and we have shown that IRF5 slows cell growth [16,18], in this particular cell line we observed no gross difference in cell proliferation or doubling time (Figure 3B & C). Therefore, while a decrease in proliferation may have been expected in IRF5-231 cells, the small difference in growth rate was not enough to explain the differences found in Figure 2. Using Boyden chambers, we individually assessed migration and invasion. In both cases, cells were plated in the upper inserts in serum-free media while the bottom chambers contained media with 10% serum as the sole chemoattractant. As seen in Figure 3D, stably expressing IRF5-231 cells gave an 80% decrease in cell migration at 4 hours. To evaulate invasiveness, a thin layer of matrigel was applied and allowed to solidify on top of the insert and cells were subsequently plated on top of the protein layer. IRF5-231 cells showed a 32% decrease in matrigel invasion (Figure 3E).

Although IRF5-231 cells were observed to have a slower rate of proliferation (6% decrease) and invasion (30% decrease), data support that the the near-complete inhibition of matrigel evasion observed (Figure 2C) is mainly due to IRF5′s inhibition of cell migration (80% decrease). To ensure that IRF5-mediated inhibition of cell migration is not unique to MDA-MB-231 cells alone, we examined MCF-7 cell migration in the presence or absence of siRNAs targeting IRF5 or overexpression of IRF5 (Figure 3F). MCF-7 breast cancer cells express a moderate amount of endogenous IRF5 and are minimally invasive [23]. As shown in Figure 3G, overexpression of IRF5 in MCF-7 cells significantly inhibited cell migration, while knockdown of IRF5 significantly increased migration. Together, these data show that IRF5 inhibits breast cancer metastasis primarily through its negative regulation of mammary epithelial cell migration.

### Amino acids 115–125 contain the functional domain required to inhibit cell migration

**Figure 4:**
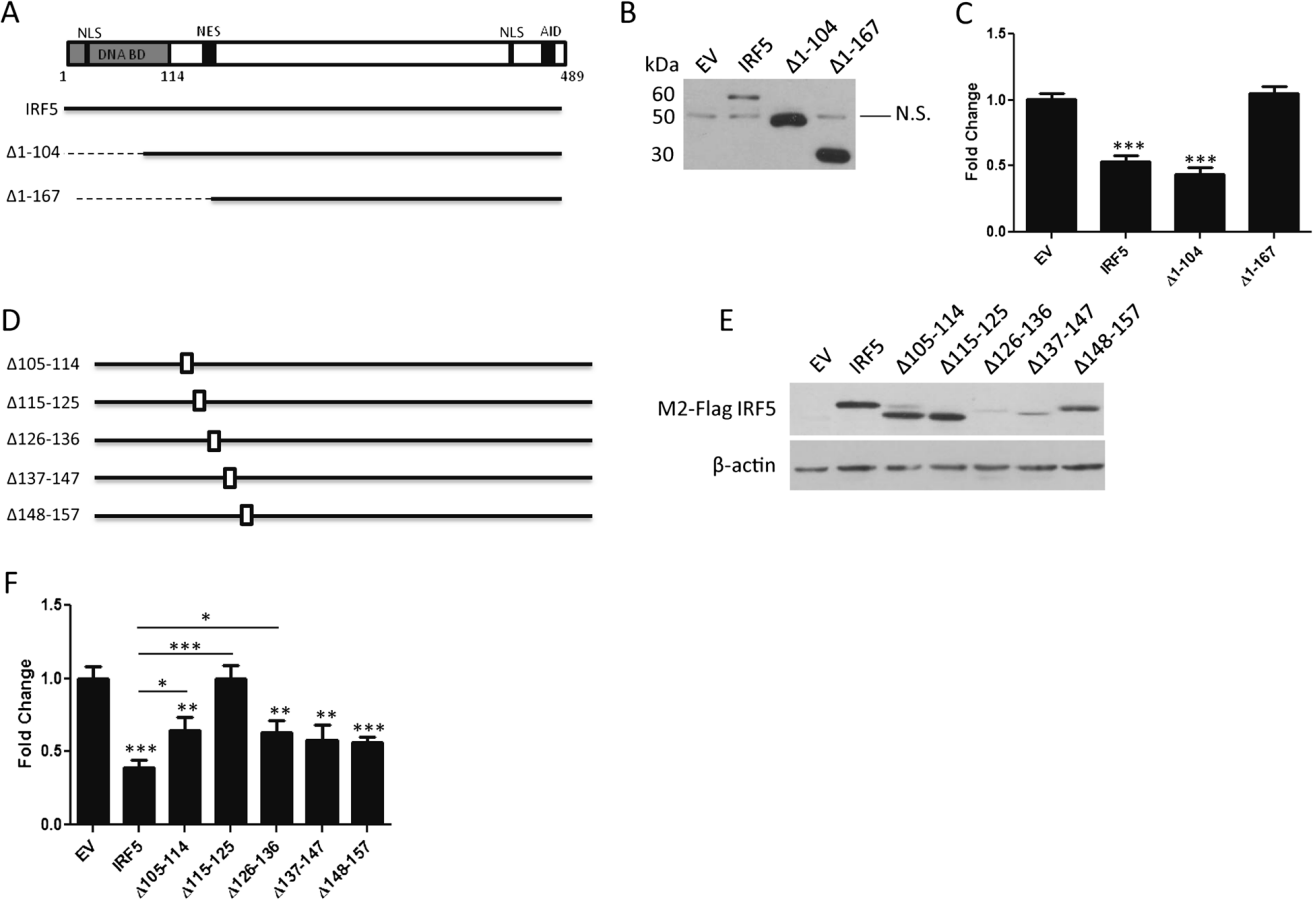
**Amino acids 115–125 are required for IRF5 to regulate cell migration. A)** Schematic representation of full-length IRF5 highlighting specific protein domains such as the DNA binding domain (DNA BD), nuclear exit signal (NES), nuclear localization signals (NLS) and autoinhibitory domain (AID). Mutants of full-length IRF5 are depicted below; dotted lines show deleted region. **B)** Expression of FLAG-tagged mutant IRF5 proteins in 231 cells. **C)** Graphical summary of data from migration assays comparing full-length IRF5 to the illustrated mutants shown in A); statistical analysis is compared to EV control. **D)** Internal deletion mutants of full-length IRF5 are shown; empty boxes indicate deleted amino acids. **E)** Expression levels of FLAG-tagged internal deletion mutants of IRF5. **F)** Similar to **C)** summarizing data from the migration assay comparing EV migratory function to full-length and mutant IRF5 function in 231 cells; statistical analysis is compared to EV control. Bars denote statistical differences between mutants and full-length IRF5. Data are from at least 3 independent experiments performed in duplicate; *p ≤ 0.05, **p ≤ 0.01, and ***p ≤ 0.0001.

Given that the regulation of cell movement by IRF5 is a novel function not previously identified, we next sought to characterize the required domain by deleting large, N-terminal amino acid regions of FLAG-tagged IRF5 as described previously [32] (Figure 4A). After sequence confirmation, these constructs were transiently transfected into MDA-MB-231 cells (Figure 4B) for use in the Boyden chamber migration assay. As shown in Figure 4C, both full-length IRF5 and the 1–104 deletion mutant (Δ1-104) show statistically significant inhibition of cell movement compared to EV-231 cells. The deletion of amino acids 1–167 (Δ1-167) or larger (Δ1-246, data not shown) no longer differed from EV-231 control cells. Furthermore, the AID domain at the C-terminus was not required for IRF5 to inhibit cell movement, as a construct containing amino acids 1–477 but lacking the AID behaved as full-length IRF5 (data not shown). This demonstrates that amino acids 105–166 of IRF5 are required for the regulation of cell movement.

To further identify the functional domain, we generated 8–10 amino acid internal deletions within the above-specified region (Figure 4D). After transient transfection (Figure 4E), migration assays were repeated. All constructs showed statistically significant inhibition of cell movement compared to EV-231 cells except for the construct containing the deletion of amino acids 115–125 (Δ115-125) (Figure 4F). Loss of function at this site indicates that the regulation of migration by IRF5 is dependent on a novel functional domain contained within amino acids 115–125. However, constructs Δ105-114 and Δ126-136 also showed statistically significant increases in migration as compared with full-length IRF5, yet not to the extent of Δ115-125 that showed identical levels of migration as EV-231. These data suggest that regions flanking amino acids 115–125 may also contribute to IRF5′s cell migration control, yet only Δ115-125 is able to fully recover the EV-231 phenotype.

### IRF5 non-transcriptionally regulates cell migration independent of its DNA binding domain and nuclear localization

**Figure 5:**
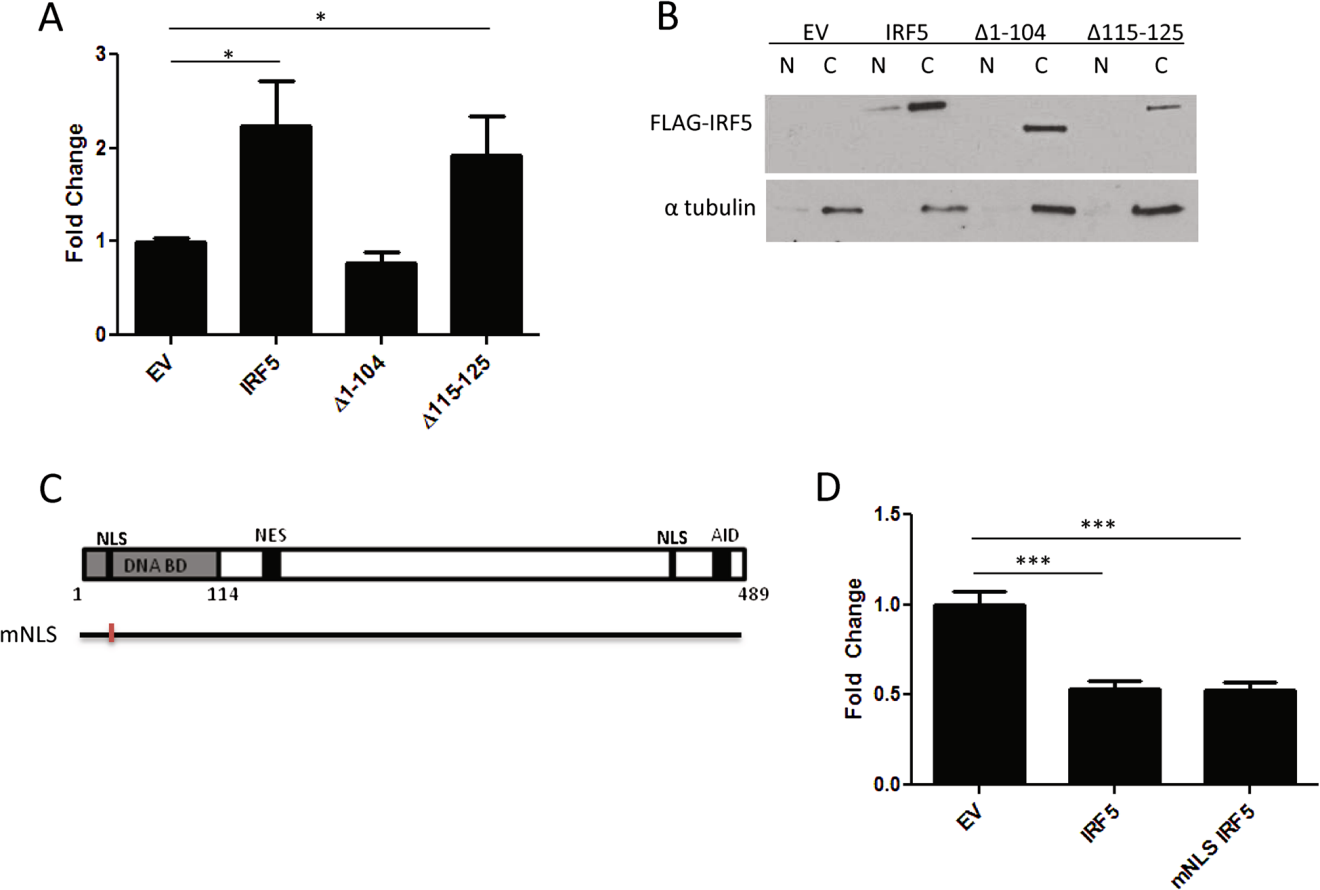
**The inhibition of cell migration by IRF5 is non-transcriptional and occurs in the cytoplasm. A)** Graphical summary of data from the dual luciferase assay measuring ISRE transactivation in the presence of full-length IRF5 or the indicated deletion mutants. Data are from 3 independent experiments performed in duplicate. **B)** Western blot analysis of cytoplasmic **(C)** and nuclear (N) extracts from 231 cells transiently transfected with the indicated plasmids. α-tubulin was used as a control for extract purity. **C)** Schematic representation of full-length IRF5 with a mutated 5′ NLS (mNLS). **D)** Graphical summary of data from the migration assay comparing EV migratory function to full-length IRF5 and the mNLS mutant in 231 cells. Data are from at least 3 independent experiments performed in duplicate; ***p ≤ 0.0001.

Since IRF5 is classically identified as a transcription factor, we next explored whether its novel role in cell migration is dependent on its transcriptional activity. The Δ1-104 mutant construct is missing its DNA binding domain, yet retains the ability to inhibit cell migration (Figure 4C). In order to confirm that the Δ1-104 construct does not bind to and transactivate reporter DNA as previously shown [2,6], and to determine whether the loss of function mutant, Δ115-125, is transcriptionally active, we performed dual luciferase assays using an ISRE luciferase promoter reporter co-transfected with either empty vector control plasmid, full-length IRF5, Δ1-104 or Δ115-125 plasmids (Figure 5A). In agreement with previously published work, full-length IRF5 increased luciferase expression as compared to EV, while Δ1-104 was unable to transactivate the reporter [2]. Importantly, the Δ115-125 mutant behaved as full-length IRF5. Together, these data indicate that IRF5′s ability to regulate cell migration is independent from its transactivation ability. This shows, for the first time, a non-transcriptional function for IRF5.

Since IRF5 controls mammary epithelial cell migration independent of its transactivation ability, we hypothesized that the function may reside within cytoplasmic molecules of IRF5. Indeed, previous studies in breast and colon adenocarcinoma models [19,23], amongst other cell types, indicate that IRF5 proteins reside in the cytoplasm of unstimulated cells. Only after stimulation with an activation trigger, such as TLR ligands or DNA damage, does IRF5 undergo post-translational modifications and translocation to the nucleus [6,7,19,22]. To confirm that the IRF5 constructs reside in the cytoplasm of unstimulated MDA-MB-231 cells, EV, full-length IRF5, Δ1-104 and Δ115-125 FLAG-tagged plasmids were transiently transfected and IRF5 cellular localization examined by immunoblot analysis of cytoplasmic and nuclear extracts. As expected, based on previously published work [6], every IRF5 construct examined remained largely in the cytoplasm (Figure 5B). Similar results were found in MCF-7 cells (data not shown). Last, to clearly discern whether small, yet undetectable amounts of nuclear IRF5 are responsible for IRF5 migratory control, an IRF5 mutant lacking the 5′ NLS of IRF5 [6] but containing the functional migratory domain (a.a. 115–125) was examined in the migration assay (Figure 5C & D). This NLS mutant was previously shown to inhibit IRF5 nuclear localization and transactivation function in unstimulated cells [6]. Data in Figure 5 clearly show that the migratory function of IRF5 is cytoplasmic and transcription-independent.

### Identification of cytoskeletal molecules that interact with IRF5 in mammary epithelial cells

**Figure 6:**
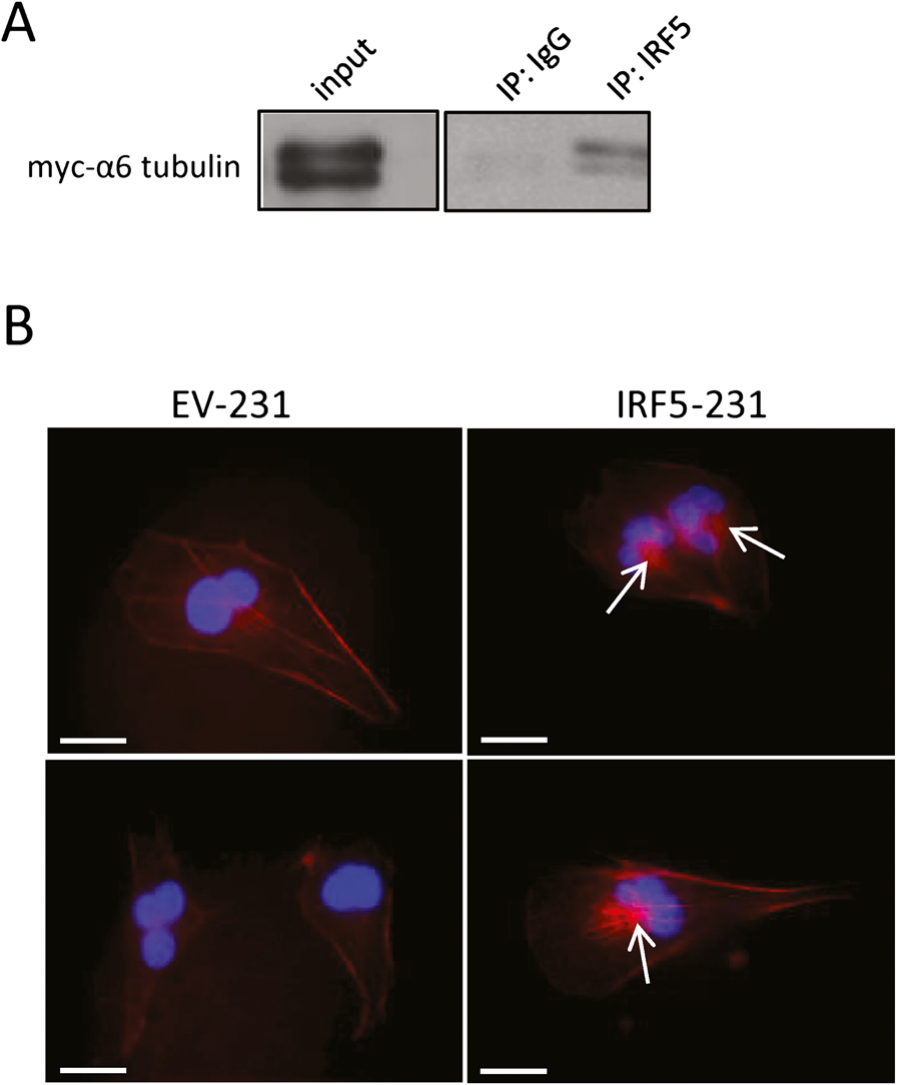
**IRF5 binds to α6-tubulin and alters F-actin bundling. A)** MCF-10A cells were transfected with myc-tagged α6-tubulin plasmid. Expression of α6-tubulin is shown on the left. Lysates were split into two equal amounts of protein for IgG control and endogenous IRF5 IP. Immunoblot from IP is shown on the right. **B)** Representative images of F-actin staining in the indicated 231 cell lines. Nuclei were counterstained with DAPI. Scale bar equals 10 μm. White arrows indicate F-actin bundling near the cell nucleus.

The cytoplasmic localization of IRF5 coupled with its non-transcriptional regulation of cell migration points to possible cytoplasmic binding partners that confer the novel migratory function to IRF5. As such, we focused our study on the identification of new IRF5 interacting partners in mammary epithelial cells. EV-231 and IRF5-231 cells were immunoprecipitated using anti-FLAG antibodies. Proteins bound to FLAG-IRF5 were identified by mass spectrometry. Several potential interacting partners of IRF5 are shown in Table 1. IRF5 had the highest ratio in IRF5/EV samples indicating that the IP was successful. Of significant interest, several of the identified proteins are known cytoskeletal molecules that have previously been implicated in breast cancer cell migration mechanics. Specifically, α6-tubulin was detected as binding 26-fold higher in IRF5-231 as compared with EV-231 control cells; filamin-C bound IRF5 4.7-fold higher, and myosin-9 (myosin IIa) 2.4-fold higher. Interaction of IRF5 with total tubulin (data not shown) and the α6 subunit of tubulin was confirmed in MDA-MB-231 cells and MCF-10A cells, respectively (Figure 6A). Given that all three of these cytoskeletal molecules have been implicated in cancer migration, invasion and metastasis formation [33], in part, through their ability to participate in filamentous (F)-actin networking that is integral for cell motility [33,34], we examined F-actin networking in EV-231 and IRF5-231 cells and found distinct and condensed F-actin bundling at the nucleus of IRF5-231 cells as compared with EV-231 cells (Figure 6B). Together, these data indicate new mechanisms to be explored by which IRF5 controls mammary epithelial cell migration.

**Table 1:** **IRF5 interacting partners in MDA-MB-231 cells**

**Identified protein**	**IRF5/EV ratio**
Interferon Regulatory Factor 5	32.9
Tubulin alpha-1C chain	26.0
Mitochondrial heat shock 60kD	7.1
Similar to Homo sapien solute carrier family 25	5.5
Similar to transketolase	5.5
Similar to EF2	4.7
Filaggrin	4.7
Fructose-bisphosphate aldolase	3.1
Similar to protein disulfide-isomerase A6	2.7
Clathrin heavy chain 2	2.7
Flap endonuclease 1	2.7
Filamin-C	2.7
Triosephosphate isomerase	2.4
Myosin-9	2.4
Ribosomal protein L12 variant	2.4
Eukaryotic translation intiation factor 3 sub. D	2.4
Mannosyl-oligosaccharide glucosidase	2.4
U4/U6 snRNA Prp31	2.4

Following immunoprecipitation of FLAG from FLAG-tagged EV- or IRF5-231 cells, IP lysate was analyzed by mass spectrometry. Binding of proteins identified was normalized to EV-231 FLAG levels. Proteins showing a greater than 2-fold increase in binding are shown.

### Global role for IRF5 in controlling epithelial cell migration

**Figure 7:**
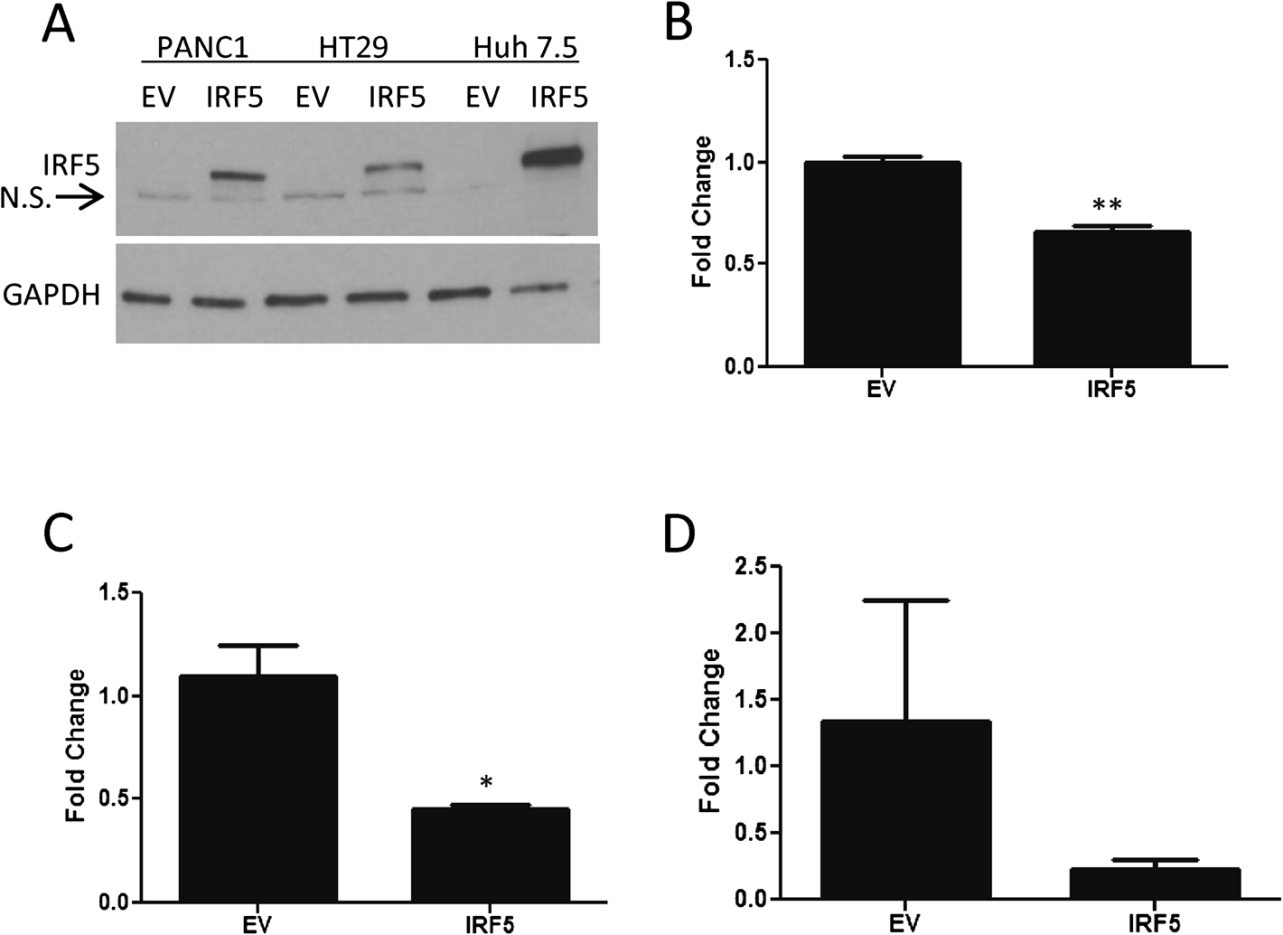
**IRF5 is a global regulator of epithelial cell migration. A)** Western blot analysis of endogenous and ectopic IRF5 expression in PANC1, HT29 and Huh 7.5 epithelial carcinoma cell lines. **B)** Graphical summary of data from the migration assay comparing EV migratory function to full-length IRF5 in PANC1 pancreatic carcinoma cells. Left panel shows fold-change; right panel shows the total number (#) of migrated cells. Data are from 3 independent experiments performed in duplicate. **C)** Same as in B) except migration of HT29 colorectal carcinoma cells was examined. **D)** Same as **B)** & **C)** except migration of Huh7.5 hepatic carcinoma cells was examined. Fold change is shown. *p ≤ 0.05, **p ≤ 0.01.

To determine whether IRF5′s control over cell migration is specific to mammary epithelial cells, we examined IRF5-mediated migration in three other epithelial cancer cell types - PANC1 human pancreatic carcinoma cells, HT29 human colon adenocarcinoma cells, and Huh 7.5 human hepatic carcinoma cells. Endogenous IRF5 expression was low to undetectable in all three carcinoma cell lines examined (Figure 7A), while relatively high levels are expressed in the normal epithelial counterpart. Similar to results obtained in migration assays with MDA-MD-231 and MCF-7 mammary epithelial cell lines (Figures 2 & 3), expression of IRF5 in PANC1 and HT29 cells resulted in significantly reduced cell migration (Figure 7B & C). Although not found to be significantly different at the 4 hr time point examined, Huh 7.5 cells expressing IRF5 gave the same trend in reduced migration (Figure 7D). These data indicate IRF5′s negative control over cell migration is not restricted to mammary epithelial cells but functions in other epithelial cell types suggesting a more global role for IRF5 as a regulator of epithelial cell migration.

## Discussion

Metastasis of primary breast cancer to distant sites and recurrence to incurable disease are the main causes of breast cancer fatalities. While migration of breast cancer cells out of a duct or lobule is a prerequisite for invasion and metastasis, the ability of these cells to migrate at all is due to intrinsic intratumoral changes, as well as extrinsic microenvironment changes. In this study, we have focused on the intrinsic intratumoral changes that occur to foster a cancer cell's metastatic potential. Here, we demonstrate that the loss of expression of a single protein, IRF5, in mammary epithelial cells enhances their migratory potential. Conversely, re-expression of IRF5 in mammary epithelial cells dramatically reduced their ability to migrate. Somewhat surprising, this newly identified function for IRF5 was not specific to mammary epithelial cells alone and appears to be functional in other epithelial cell types. Data presented herein not only identify a new cellular function for the transcription factor IRF5, but the first, non-transcriptional and cytoplasmic function for IRF5.

Although IRF5 is primarily known as a transcriptional immune regulator [2,4,6,11,13,15], our lab and others have shown that it is also a critical regulator of DNA damage-induced apoptosis [8,19,21] and expression of IRF5 is lost in a variety of cancers [16,19,23-27] supporting an important tumor suppressor role for IRF5. We became very interested in understanding the functional consequence of loss of IRF5 expression in mammary epithelial cells when we found that normal human breast epithelium expresses high levels of IRF5 and expression decreases as malignancy progresses from non-metastatic DCIS to IDC [23]. Not surprising given its known immunoregulatory functions, recent data also support an important extrinsic role for IRF5 in human breast cancer progression through its ability to regulate the tumor-immune microenvironment [35]. Based on our original finding, however, that IRF5 alone could revert the invasive outgrowth of MDA-MB-231 cells in 3D [23], we hypothesized that its expression could also inhibit intrinsic cellular processes that would otherwise enhance the metastatic potential of mammary epithelial cells. Indeed, we found by the wound healing assay, matrigel invasion assay, and standard Boyden chamber migration and invasion assays that IRF5 expression abrogated cell movement (Figures 2 & 3). Independent analysis of cell proliferation, viability, motility and invasion revealed that IRF5 had the most significant effect on cell migration. Although less effective in the cell invasion assays, previous data support that IRF5 may control invasion through a mechanism involving the dysregulation of matrix metalloproteinases (MMPs) [23]. Nonetheless, even in the non-invasive MCF-7 cell line, IRF5 regulated cell migration in a dose-dependent manner, where over expression of IRF5 slowed cell migration and knockdown increased migration (Figure 3G). This is the first time that IRF5 has been implicated in the regulation of cell migration.

Given that this is a newly identified function for IRF5, we generated a number of deletion mutants to determine the domain within IRF5 that regulates the migratory function. We identified the functional domain regulating cell migration to be within amino acids 115–125 (Figure 4F) containing the sequence: Y K I Y E V C S N G P. Of significant interest, this amino acid sequence is only found in IRF5 and not any other IRF family members, indicating that the regulatory role for IRF5 in cell migration is likely specific to IRF5. No other IRF family members have been found to have this function. Also important is the fact that this sequence is highly conserved across all species, further highlighting the importance of this functional domain within IRF5. This small region lies in between the DBD and the beginning of the PEST domain of IRF5. Very little is known about this region of the IRF5 protein to date. Further, the folding of this particular portion of the protein has not yet been visualized, since the crystal structure of dimeric IRF5 has only been examined using residues 222–467 [36]. However, using the ProteinPredict Server [37] to analyze a larger sequence (115–150) containing amino acids 115–125, we found that this particular region is predicted to form a loop that is mostly exposed to solvent and potential interacting partners. Additional analysis using a BLAST search of the short (115–125) and long (115–160) amino acid sequences that encode for the IRF5 functional domain identified several varied proteins with similar amino acid stretches, but with no functional similarity found between them. Based on this cursory assessment, the physical structure of this region is needed to elucidate more detailed functional features.

Another striking observation to come from the current study is that the identified migratory domain of IRF5 is not dependent on its transcription factor function. We confirmed that IRF5 transactivation ability is lost in the mutant construct missing its DBD (Δ1-104, Figure 5A) but that it is still able to inhibit cell migration (Figure 4C), whereas the internal deletion mutant (Δ115-125) retains its ability to transactivate the ISRE promoter (Figure 5A) but has lost its cell migratory function (Figure 4F). Based on these data and previous work on the mechanisms controlling IRF5 cellular localization [6,7,19,38], we hypothesized that this new migratory function of IRF5 must be cytoplasmic. Indeed, others and we have already shown that IRF5 is primarily cytoplasmic in the majority of cell lines examined, including mammary epithelial cells [19,23], and that nuclear translocation requires activation via post-translational modification [6,7,10,14,19,38]. We show by immunoblot analysis of cyto/nuclear extracts that IRF5 and the ∆115-125 mutant are cytoplasmic (Figure 5B). Furthermore, using a previously generated NLS mutant of IRF5 that retains IRF5 in the cytoplasm of unstimulated cells [6], we show unambiguously that IRF5′s migratory function is cytoplasmic (Figure 5D). Taken together, these data demonstrate for the first time a non-transcriptional and cytoplasmic role for IRF5 in mammary epithelial cell migration.

Although the molecular mechanism(s) by which IRF5 regulates cell migration has not been fully elucidated, we identified potential IRF5 cytoplasmic interacting partners to gain further insight into the mechanisms of IRF5 migratory function. As shown in Table 1, several novel interacting partners were identified, some of which are already known cytoskeletal proteins that regulate cell migration. The protein found to predominantly bind to IRF5 in this model was α6-tubulin and we independently confirmed interaction of endogenous IRF5 with α6-tubulin in MCF-10A cells (Figure 6A). Tubulin is a constituent of microtubules, which are a major player in the mechanics of cell movement. During cell migration, tubulin elongates and shortens as required [39,40]. This process is mediated by the stability of the growing, or positive ends, of the microtubule. Rho GTPases Rac1 and Cdc42 increase the stability of the positive ends, allowing for growth of the microtubule [41]. Not surprisingly, Rac1 and Cdc42 activity are often increased in highly motile cells and metastatic cancer [41]. Data in non-motile MCF-10A cells indicate that phosphorylation of serine 165 of α6-tubulin by PKCα is sufficient to cause an increase in MCF-10A cell migration and levels of PKCα activity are high in MDA-MB-231 cells [41]. Filamin and myosin are two other cytoskeletal proteins identified to potentially interact with IRF5. Filamins contribute to cell movement by binding to actin proteins, such as filamentous (F)-actin, and serve as scaffolds. Rho GTPases Rac1 and Cdc42 interact with Filamins [42]. Nonmuscle myosin is an actin-based motor protein essential to cell motility, cell division, migration, adhesion, and polarity. Myosin IIa is involved in light and heavy chain assembly in breast cancer cell lines [43,44] and depletion via small interfering RNA impairs migration. Given that all three of these cytoskeletal proteins have been implicated in F-actin networking [33], it is tempting to speculate that IRF5 most likely regulates epithelial cell migration- at least in part- via its functional interaction with one or more of these binding partners. Indeed, data in Figure 6B suggest that IRF5 may control cell migration/motility by inducing the shortening or contraction of F-actin fibers resulting in bundling at the F-actin cortex near the nucleus and/or inducing altered “retrograde flow” where the F-actin network is transported from the leading edge of a cell towards the cell center [33,45]. Either of these events would inhibit or slow down cell migration/motility [46]. Thus, efforts are now focused on the further examination of IRF5 migratory function in other epithelial cell types and determining whether IRF5 regulates cell migration by altering mechanisms of F-actin networking.

## Conclusions

We have identified for the first time a non-transcriptional and cytoplasmic function for IRF5 that inhibits breast cancer cell migration. We identified a new domain responsible for this function residing within amino acids 115–125 of IRF5 that is conserved across species and not present within other IRF family members. Notably, we found that IRF5′s control of cell migration is not restricted to mammary epithelial cells but functions in other epithelial cell types suggesting a more global role for IRF5 in cell migration. Thus, these findings are significant as they identify a new regulator of epithelial cell migration and suggest that the observed loss of IRF5 expression/function in breast cancer may also be common among other epithelial-derived malignancies as a mechanism to increase metastatic potential.

## Material and methods

### Cell lines and culture

MCF-10A, MCF-7, and MDA-MB-231 human breast cells were purchased from American Type Culture Collection (ATCC; Manassas, VA, USA) and cultured as described previously [23]. PANC1 human pancreatic carcinoma cells and HT29 human colon adenocarcinoma cells were obtained from Ms. Anita Antes in the NJMS Department of Biochemistry & Molecular Biology tissue culture facility. Cells were cytogenetically tested and authenticated (by STR profiling from ATCC) before freezing. The amphotrophic helper-free PLAT-A cells were provided by U. Herbig (Rutgers, NJ, USA) and cultured as previously described [20].

### Retroviral transduction and transient transfection

Stable cell lines from batch cultures expressing IRF5 were made as previously described using the pBabe-IRF5-puromycin plasmid [23]. Transient transfections were carried out with 2 μg of same plasmid DNA using either the Lipofectamine 2000 (Invitrogen) transfection reagent or 4D Nucleofector SF Cell Line Kit (Lonza-Amaxa) according to manufacturer's instructions. IRF5 siRNA knockdown experiments were performed using the 4D Nucleofector SF Cell Line kit with 150–300 ng of siRNA (Fisher). Cells were used for experiments 48–72 hours following transfection.

### Molecular constructs

The pCMV-Tag2B IRF5 variant 3/4 mammalian expression plasmids and IRF5 5′-nuclear localization signal (5′mNLS) mutant plasmid were previously described [2,32]. FLAG-tagged amino and carboxyl terminal deletion mutants as well as internal deletion mutants of IRF5 were constructed as described by Korczeniewska et al. [32]. Briefly, mutants were generated by PCR amplification of the full-length IRF5 template with *Pfu* Turbo DNA polymerase (Stratagene) using the Quick Change Lightening site-directed mutagenesis kit protocol (Aligent). All construct sequences were verified by DNA sequencing (Macrogen) and protein expression confirmed by Western blot analysis with anti-Flag M2 antibodies (Sigma-Aldrich, Cell Signaling) or anti-IRF5 antibodies (Cell Signaling, Novus). The firefly luciferase *ISRE* promoter reporter and Renilla luciferase plasmids were previously described by Korczeniewska et al. [32].

### Western blots and immunoprecipitations

Whole cell lysates were prepared in GST-FISH lysis buffer (10% glycerol, 50 mM Tris pH 7.4, 0.1 M NaCl, 1% NP-40 and 2 mM MgCl_2_) containing 0.2 mM protease inhibitor cocktail (Sigma-Aldrich). Cytoplasmic extracts were prepared by washing cells twice with cytoplasmic extract wash buffer (10 mM Hepes, 10 mM KCl, 0.2 mM protease inhibitor cocktail). Cell pellets were resuspended in cytoplasmic extract buffer (10 mM Hepes, 10 mM KCl, 1% NP-40, protease inhibitor cocktail) and incubated at 4°C for 10 minutes on a rocker. Supernatant was removed as cytoplasmic extract and lysate buffer (20 mM Hepes, 20% glycerol, 500 mM KCl, 0.2 mM EPTA, and 1.5 mM MgCl2, protease inhibitors) added. The remaining nuclear pellet was then resuspended in lysate buffer and rocked at 4°C for 15 minutes. Nuclei were spun down at max speed for 10 minutes and the nuclear extract supernatant collected. For immunoprecipitation, FLAG-tagged IRF5 was immunoprecipitated with anti-FLAG M2 affinity beads (Sigma-Aldrich). 10% SDS PAGE gels were used to resolve proteins, which were then transferred to nitrocellulose membranes (BioRad). Anti-FLAG or anti-IRF5 (Novus, Cell Signaling) primary antibodies were added to the membrane at a 1:1000 dilution and later probed with either anti-mouse or anti-rabbit horseradish peroxidase (HRP)-conjugated secondary antibodies (Cell Signaling) at a 1:2000 dilution. Cytoplasmic purity was confirmed with anti-α-tubulin antibodies (Cell Signaling). Blots were visualized with enhanced chemiluminescence using ECL Western Blotting detection reagents (Amersham Biosciences). Equal loading was confirmed by the expression of β-actin or GAPDH (Cell Signaling).

### Wound healing assay

0.5 × 10^6^ cells were plated in each well of a 6-well plate with 2 ml of complete media. Cells were allowed to grow until confluent when a disruption in the monolayer was created with a sterile p-10 pipette tip. Cells were grown in serum-free media until the end of the experiment. Wells were rinsed with serum-free media four times to remove cell debris before imaging the same area at specified time points. Wounds were measured by width at 3 points and averaged.

### Matrigel evasion assay

The matrigel drop assay was described previously [31] and modified by suspending 1,000 cells in 5 μL of complete media and combining the cell suspension with 5 μL of thawed Growth factor-reduced basement membrane matrix (matrigel; BD Pharminigen). The cell/matrigel suspension was plated as a drop in the center of a 6-well plate and placed in the incubator for 5 minutes to solidify. 2 ml of media containing 5% serum was placed over the matrigel drop and cells were observed at specified time points. The number of cells migrating out of the drop and distance of migration were measured.

### Cell proliferation and viability assays

To assess the rate of cell growth a colorimetric MTT-based (3-[4,5-dimethylthiazol-2-yl]-2,5-diphenyl tetrazolium bromide) assay was performed using the Cell Proliferation Kit I (Roche) according to the manufacturer's instructions. To measure absorbance, the Synergy HT plate reader and KC4 v3.4 software (Bio-Tek, Potton) were used. Absorbance of blank samples containing only media and MTT kit reagents was subtracted from sample raw data to obtain actual absorbance. For cell viability, cells were stained with trypan blue and viability measured on a *Vi-Cell* (Beckman Coulter).

### Cell migration and invasion assays

Boyden chamber experiments were performed using transwell inserts containing 8.0 μm pores on a polycarbonate membrane in 24 well plates (Corning) to assess cell migration and invasion. 48 hours post-transfection, cells were serum-starved overnight before use. For the invasion assay, 100 μl of 230 μg/ml matrigel diluted in serum free DMEM was placed on the top of each transwell insert and left overnight in the incubator to solidify. On the day of the experiment, 0.1 × 10^6^ cells/insert were plated in 200 μl serum free media into the top chamber and 650 μl of 10% FBS DMEM was added to the bottom chamber. For cell migration, cells were allowed to migrate through the membrane for 4 hours in the incubator. For cell invasion, cells were placed in the incubator for 16 hours. At the end of the assay, each insert was washed 3 times in cold PBS (137 mM NaCl, 2.7 mM KCL, 4.3 nM Na_2_HPO_4_x7H_2_0, 1.4 mM KH_2_PO_4_) and PBS-soaked Q-tips were used to clean the top of each insert 3 times. To fix cells, inserts were placed in 650 μl of ice cold 100% methanol at -20°C for 10 minutes. Inserts were then placed into the same volume of 0.2% crystal violet in 50% methanol at room temperature for 10 minutes to stain. Each insert was rinsed in ddH_2_0 3 times and air dried over night upside down. Using a scalpel, the membranes were cut out of the insert and mounted cell-side down onto glass slides with Polymount hard-set mounting medium (Polysciences, Inc). To quantitate cell movement, cells in 5 random high power fields of view (400×) from each membrane were counted and averaged. Results were normalized to control as fold change.

### ISRE promoter reporter assay

48 hours following MDA-MB-231 transient transfection in a 6-well plate with 1 μg of ISRE luciferase reporter plasmid, 2 μg of either pCMV.2b EV, IRF5 or ΔDBD IRF5 and 20 ng of pRL, promoter activity was determined using the Dual-Luciferase Reporter Assay System (Promega) according to the manufacturer's instructions. Luminescence was measure on a Packard LumiCount plate reader and analyzed with Packard Plate Reader Version 3.0 software. Levels of reporter firefly luciferase activity were normalized to Renilla luciferase activity and fold change was attained by normalizing to the values of an empty vector control.

### Immunoflourescence microscopy

For F-actin staining, EV-231 and IRF5-231 cells were grown on glass microscope slides, washed in PBS and fixed in a 4% paraformaldehyde PBS solution for 20 minutes at room temperature. Cells were then permeabilized for 20 minutes in PBS containing 0.2% Triton X-100. After blocking, slides were incubated with a 1:100 dilution of phalloidin stain, mounted with DAPI aqueous mounting medium and images taken at 60× magnification on a Zeiss Axiovert 200 M microscope and analyzed using Axiovision version 4.8 software.

### Reversed Phase Liquid Chromatography Mass Spectrometry analysis (RPLC-MS)

Proteins were eluted from anti-FLAG M2 affinity beads by boiling in Laemmli sample buffer. Eluted proteins were then run on 12% polyacrylamide gels. After staining the gel with Coomassie Brilliant Blue, protein gel bands were diced into 1 mm3 pieces and washed with 30% acetonitrile in 50 mM ammonium bicarbonate before DTT reduction and iodoacetamide alkylation. Trypsin was used for digestion at 37°C overnight. The resulting peptides were extracted with 30 μl of 1% trifluoroacetic acid followed by C18 ziptip desalting. Peptides were further fractionated by RPLC on an Ultimate 3000 LC system (Dionex, Sunnyvale, CA) coupled with a Q Exactive mass spectrometer (Thermo Scientific) via a Thermo Scientific nano electrospray ionization source. The mass spectrometer was operated in a Top 15 data dependent mode with automatic switching between MS and MS/MS. Source ionization parameters were as follows: spray voltage: 2.2 kV; capillary temperature: 275°C, s-lens: 50.0. Full scan MS mode (300–1650 m/z) was operated at a resolution of 70,000, automatic gain control (AGC) target; 1 × 10^6^, maximum ion transfer time (IT): 500 ms. Ions selected for MS/MS were subjected to the following parameters: resolution: 17, 500, AGC: 5 × 10^4^, IT 250 ms; 4.0 m/z isolation window; normalized collision energy 25.0; underfill ratio 5.0%; and dynamic exclusion: of 30.0 s.

### Mass spectrometric database search

Each of the raw files was analyzed using the Thermo Proteome Discoverer (Ver 1.3) platform with Mascot (2.4.1) as search engine against the human protein sequences of nonredundant Uniprot protein database. The following Mascot parameters were used: trypsin, two missed cleavages, precursor mass tolerance: 10 ppm, fragment mass tolerance: 0.1 Da, dynamic modifications: methionine oxidation and carbamidomethylation of cysteine. Decoy search option for Mascot was engaged. Scaffold (version Scaffold_4.2.1, Proteome Software Inc., Portland, OR) was used to validate MS/MS based peptide and protein identifications. Protein quantification was performed using spectral counting method which involves summing the total number of tandem mass spectra that are detected and identified for a given protein in respective samples. Fold ratio was calculated by dividing spectral count values from IRF5 sample by that of the empty vector sample.

### Statistical analysis

Experimental data is presented as the mean +/− the standard deviation from at least three independent experiments performed in duplicate. Differences between groups were analyzed by the Student's *t*-test. Statistical analysis was performed using Prism 4.0 (GraphPad Prism 5 Software). Statistical significance is defined as *p ≤ 0.05, **p ≤ 0.01, and ***p ≤ 0.0001.
